# Supplementary material for: Inhibitors of 3-Hydroxy-3-methylglutaryl Coenzyme A Reductase Decrease the Growth, Ergosterol Synthesis and Generation of petite Mutants in Candida glabrata and Candida albicans
Source: Int J Mol Sci. 2023 Nov 28;24(23):16868. doi: 10.3390/ijms242316868 (PMC10706242; doi:10.3390/ijms242316868)
Supplement: Supplementary file 1 [file ijms-24-16868-s001.zip › ijms-2716590-supplementary.pdf]

Supplementary Materials

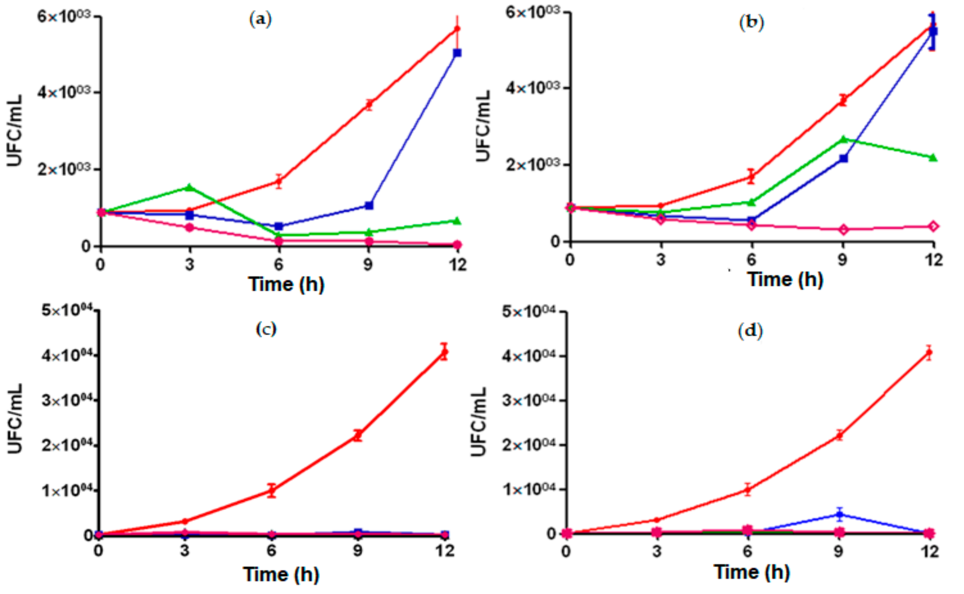

Figure S1. Growth inhibition of *C. glabrata* (a-b) and *C. albicans* (c-d) with simvastatin and fluconazole. The yeasts (2x10^4 cells/mL) were grown in absence (red lines) and presence of 100 (blue lines), 300 (green lines) and 600 µM (fushia lines) of simvastatin (a,c) and fluconazole (b,d) for 3, 6, 9 and 12 h at 37 °C. After treatment, they were inoculated on YPD agar plates and incubated for 24 h to determine the number of UFC/mL.

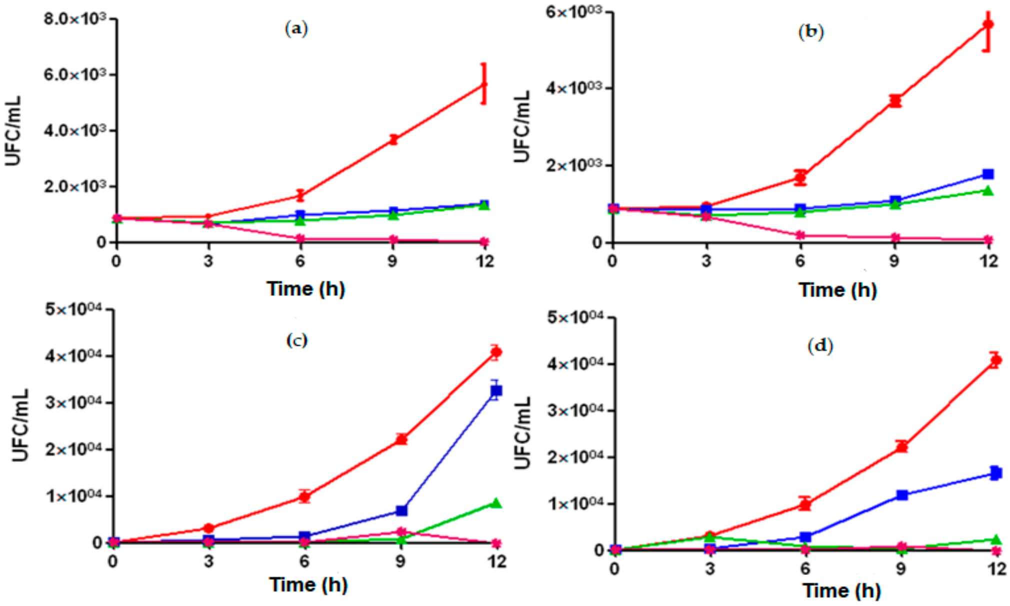

Figure S2. Growth inhibition of *C. glabrata* (a-b) and *C. albicans* (c-d) with 1a and 1b. The yeasts (2x10^4 cells/mL) were grown in absence (red lines) and presence of 100 (blue lines), 300 (green lines) and 600 µM (fushia lines) of 1a (a,c) and 1B (b,d) for 3, 6, 9 and 12 h at 37 °C. After treatment, they were inoculated on YPD agar plates and incubated for 24 h to determine the number of UFC/mL.

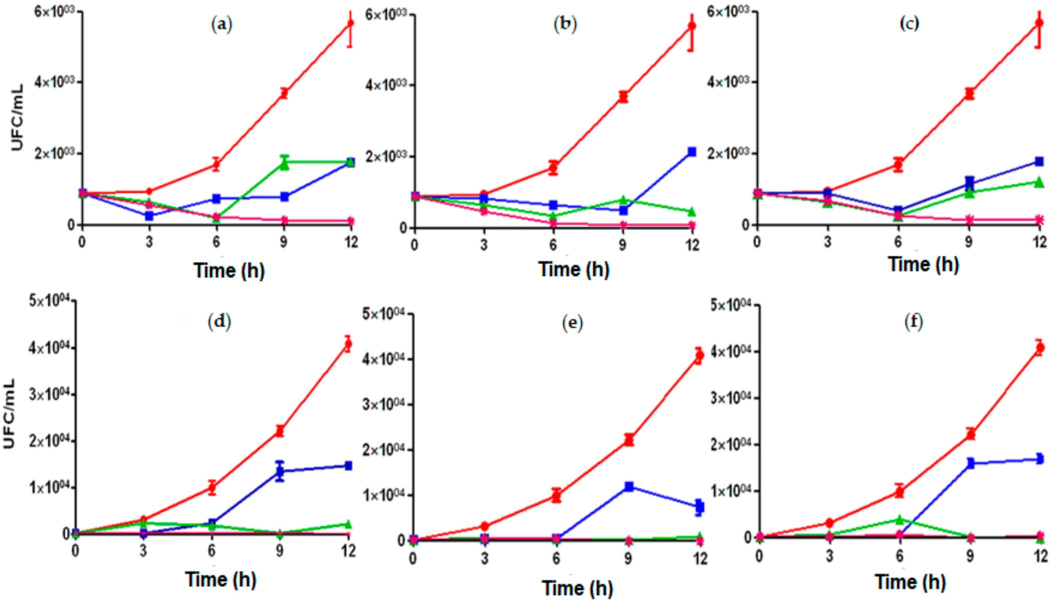

Figure S3. Growth inhibition of *C. glabrata* (a-c) and *C. albicans* (d-f) with 2a, 2b and 2c. The yeasts (2x10^4 cells/mL) were grown in absence (red lines) and presence of 100 (blue lines), 300 (green lines) and 600 μM (fushia lines) of 2a (a,d), 2b (b,e) and 2c (c,f) for 3, 6, 9 and 12 h at 37 °C. After treatment, they were inoculated on YPD agar plates and incubated for 24 h to determine the number of UFC/mL.

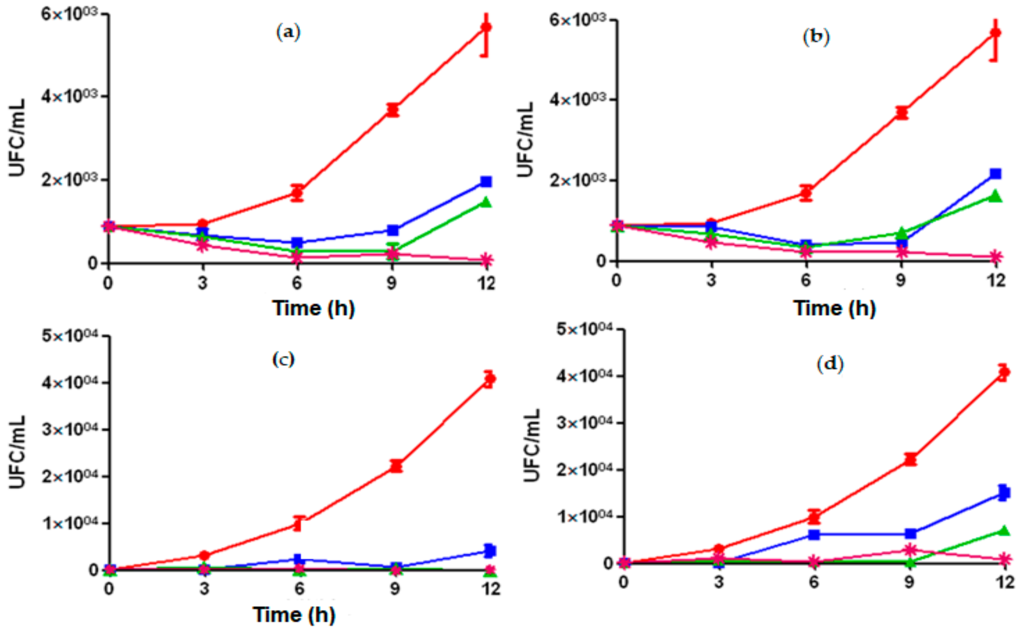

Figure S4. Growth inhibition of *C. glabrata* (a-b) and *C. albicans* (c-d) with 3a and 3b. The yeasts (2x10^4 cells/mL) were grown in absence (red lines) and presence of 100 (blue lines), 300 (green lines) and 600 μM (fushia lines) of 3a (a,c) and 3b (b,d) for 3, 6, 9 and 12 h at 37 °C. After treatment, they were inoculated on YPD agar plates and incubated for 24 h to determine the number of UFC/mL.

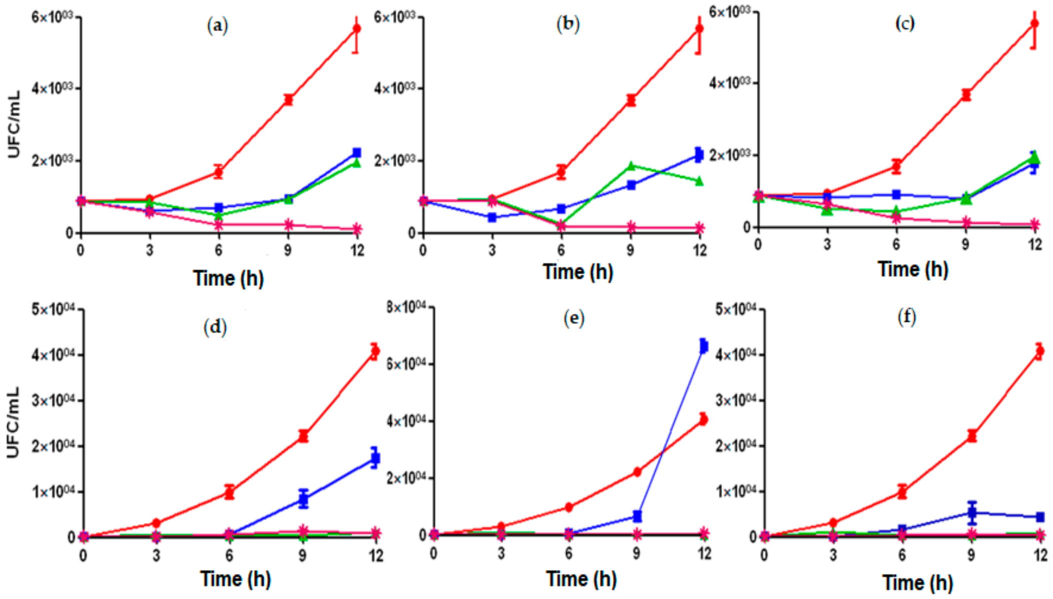

Figure S5. Growth inhibition of *C. glabrata* (a-c) and *C. albicans* (d-f) with 4a, 4b and 4c. The yeasts (2x10[4] cells/mL) were grown in absence (red lines) and presence of 100 (blue lines), 300 (green lines) and 600 µM (fushia lines) of 4a (a,d), 4b (b,e) and 4c (c,f) for 3, 6, 9 and 12 h at 37 °C. After treatment, they were inoculated on YPD agar plates and incubated for 24 h to determine the number of UFC/mL.

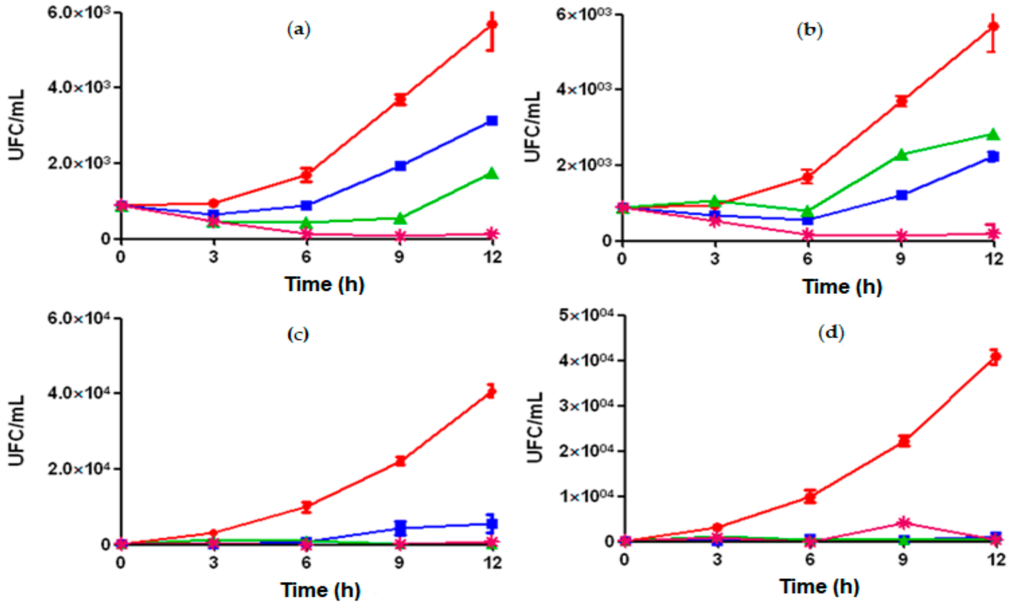

Figure S6. Growth inhibition of *C. glabrata* (a-b) and *C. albicans* (c-d) with 5a and 5b. The yeasts (2x10[4] cells/mL) were grown in absence (red lines) and presence of 100 (blue lines), 300 (green lines) and 600 µM (fushia lines) of 5a (a,c) and 5b (b,d) for 3, 6, 9 and 12 h at 37 °C. After treatment, they were inoculated on YPD agar plates and incubated for 24 h to determine the number of UFC/mL.

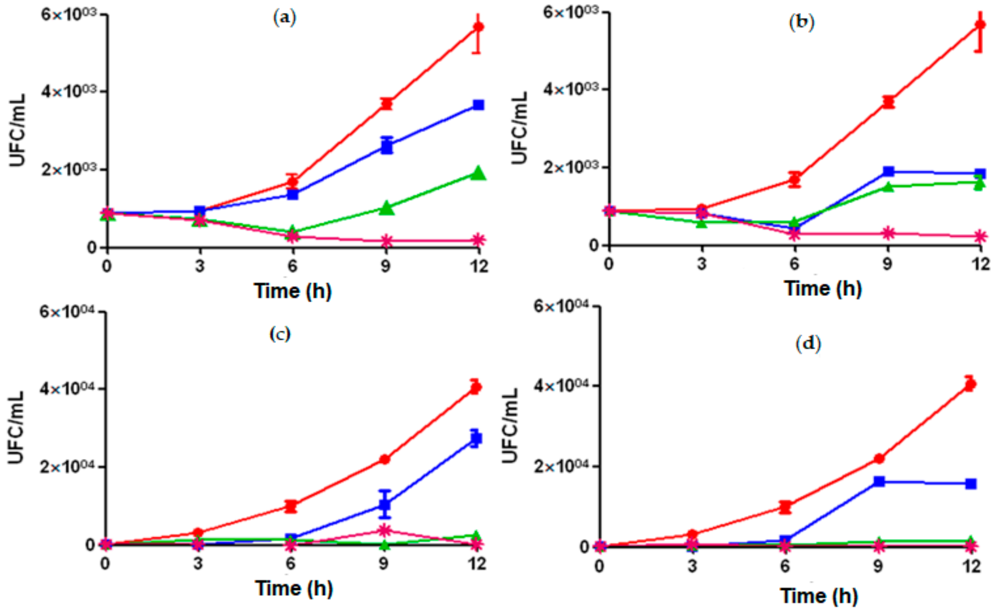

Figure S7. Growth inhibition of *C. glabrata* (a-b) and *C. albicans* (c-d) with 6a and 6b. The yeasts (2x10^4 cells/mL) were grown in absence (red lines) and presence of 100 (blue lines), 300 (green lines) and 600 µM (fushia lines) of 6a (a,c) and 6b (b,d) for 3, 6, 9 and 12 h at 37 °C. After treatment, they were inoculated on YPD agar plates and incubated for 24 h to determine the number of UFC/mL.

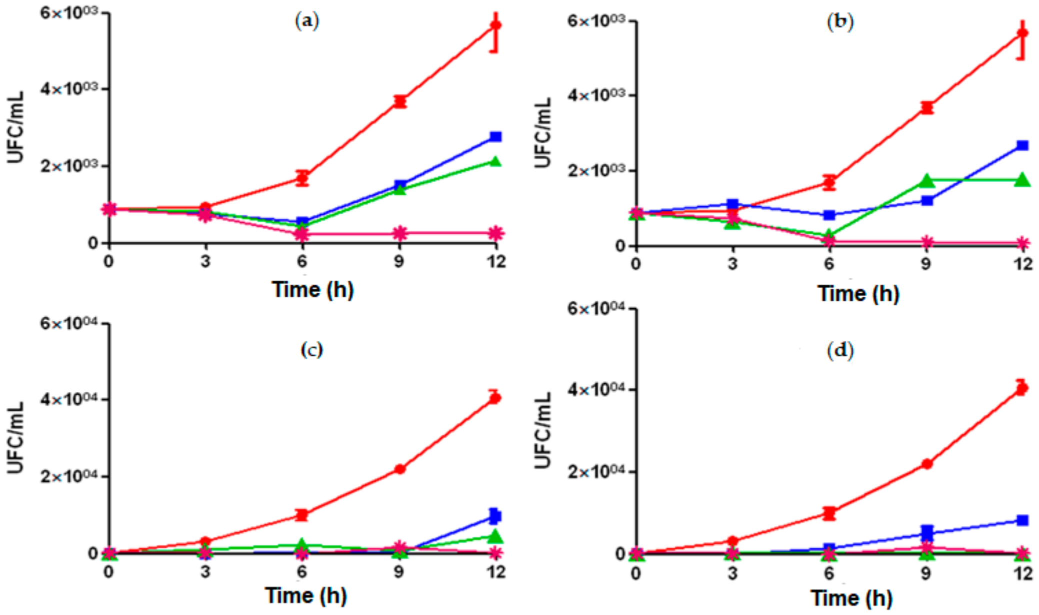

Figure S8. Growth inhibition of *C. glabrata* (a-b) and *C. albicans* (c-d) with 6c and 6d. The yeasts (2x10^4 cells/mL) were grown in absence (red lines) and presence of 100 (blue lines), 300 (green lines) and 600 µM (fushia lines) of 6c (a,c) and 6d (b,d) for 3, 6, 9 and 12 h at 37 °C. After treatment, they were inoculated on YPD agar plates and incubated for 24 h to determine the number of UFC/mL.

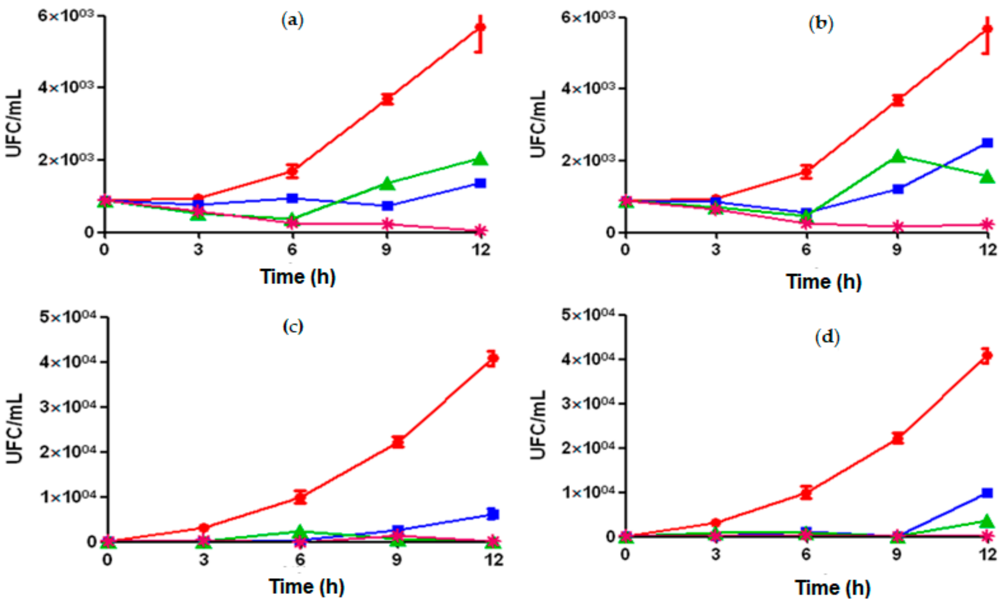

Figure S9. Growth inhibition of *C. glabrata* (a-b) and *C. albicans* (c-d) with 6e and 6f. The yeasts (2x10[4] cells/mL) were grown in absence (red lines) and presence of 100 (blue lines), 300 (green lines) and 600 µM (fushia lines) of 6e (a,c) and 6f (b,d) for 3, 6, 9 and 12 h at 37 °C. After treatment, they were inoculated on YPD agar plates and incubated for 24 h to determine the number of UFC/mL.

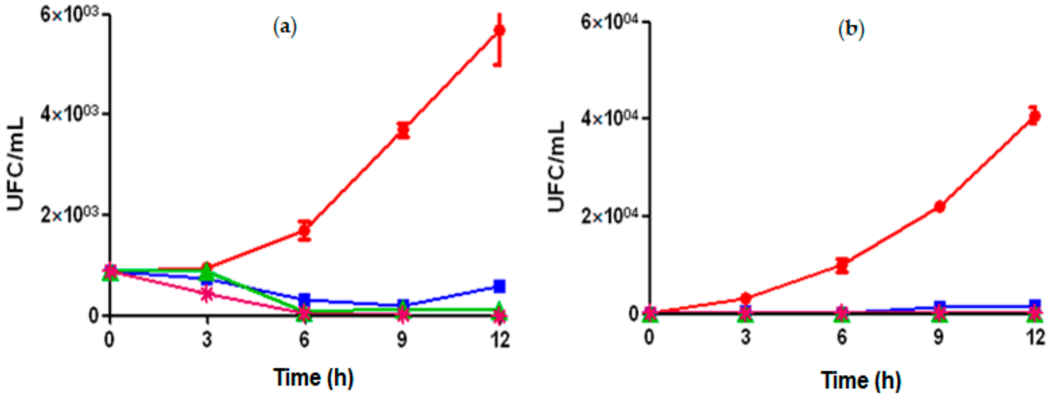

Figure S10. Growth inhibition of *C. glabrata* (a) and *C. albicans* (b) with 6g. The yeasts (2x10[4] cells/mL) were grown in absence (red lines) and presence of 100 (blue lines), 300 (green lines) and 600 µM (fushia lines) of 6g (a,b) 12 h at 37 °C. After treatment, they were inoculated on YPD agar plates and incubated for 24 h to determine the number of UFC/mL.

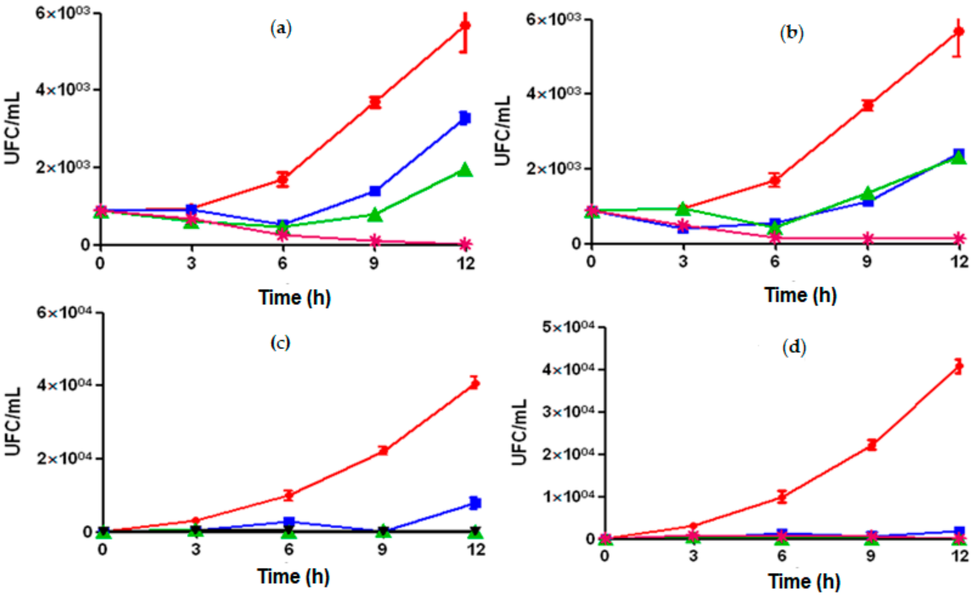

Figure S11. Growth inhibition of *C. glabrata* (a-b) and *C. albicans* (c-d) with 7a and 7b. The yeasts (2x10[4] cells/mL) were grown in absence (red lines) and presence of 100 (blue lines), 300 (green lines) and 600 μM (fushia lines) of 6e (a,c) and 6f (b,d) for 3, 6, 9 and 12 h at 37 °C. After treatment, they were inoculated on YPD agar plates and incubated for 24 h to determine the number of UFC/mL.

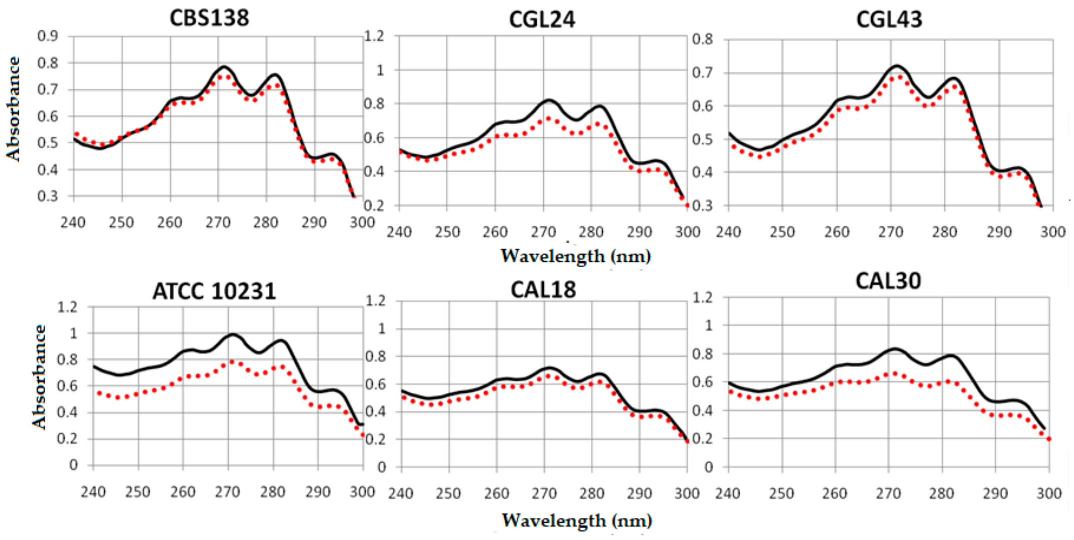

Figure S12. Reduction in ergosterol levels with simvastatin. The strains of *C. glabrata* (CBS, CGL24 and CGL43) and *C. albicans* (ATCC10231 CAL18 and CAL30) were grown in YNB without simvastatin (black line) and with 100 μM of simvastatin (res dashed line). Followed by ethanolic extraction with KOH, the fraction corresponding to sterols was scanned from 240 to 300 nm. The quantities taken correspond to equal cell mass. The graph shows the absorbance values on the "x" axes and the wavelengths (nm) in the absorption spectrum on the "y" axes.

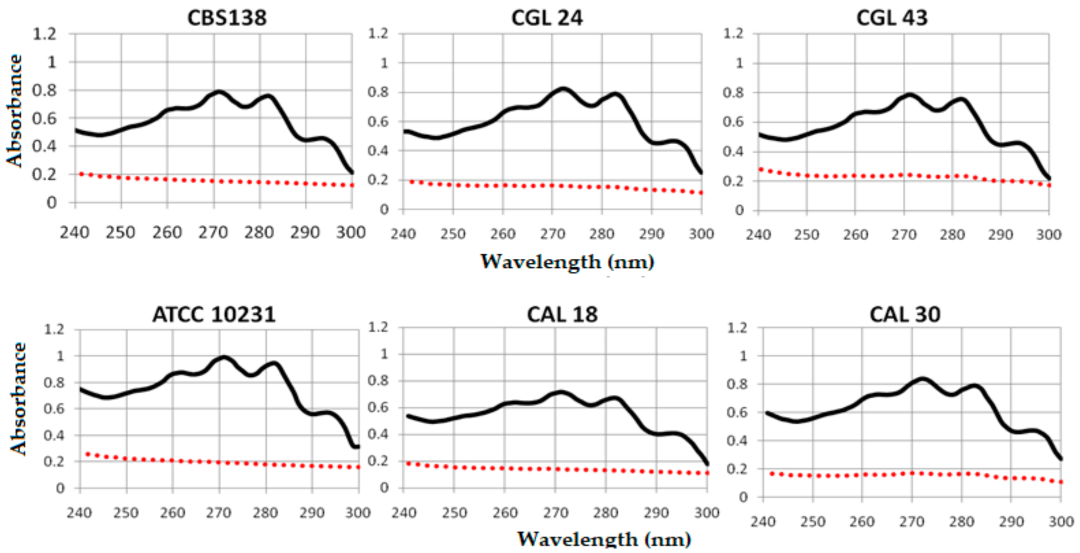

Figure S13. Reduction in ergosterol levels with simvastatin. The strains of *C. glabrata* (CBS, CGL24 and CGL43) and *C. albicans* (ATCC10231 CAL18 and CAL30) were grown in YNB without fluconazole (black line) and with 100 µM of fluconazol (red dashed line). Followed by ethanolic extraction with KOH, the fraction corresponding to sterols was scanned from 240 to 300 nm. The quantities taken correspond to equal cell mass.

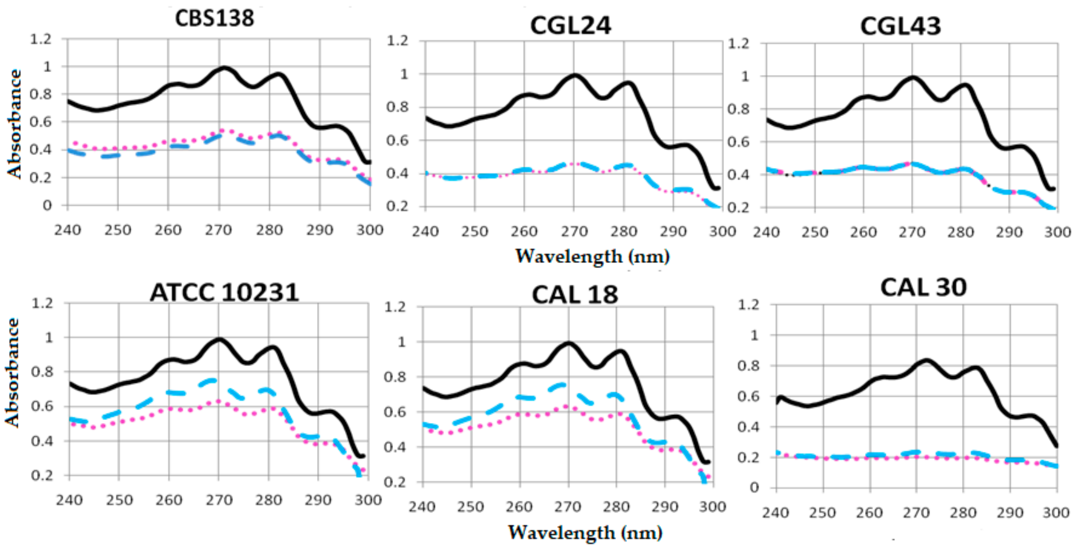

Figure S14. Reduction in ergosterol levels with simvastatin. The strains of *C. glabrata* (CBS, CGL24 and CGL43) and *C. albicans* (ATCC10231 CAL18 and CAL30) were grown in YNB without compound (black line) and with 100 µM of 1a (fushia dashed line) and of 1b (blue dashed line). Followed by ethanolic extraction with KOH, the fraction corresponding to sterols was scanned from 240 to 300 nm. The quantities taken correspond to equal cell mass. The graph shows the absorbance values on the "x" axes and the wavelengths (nm) in the absorption spectrum on the "y" axes.

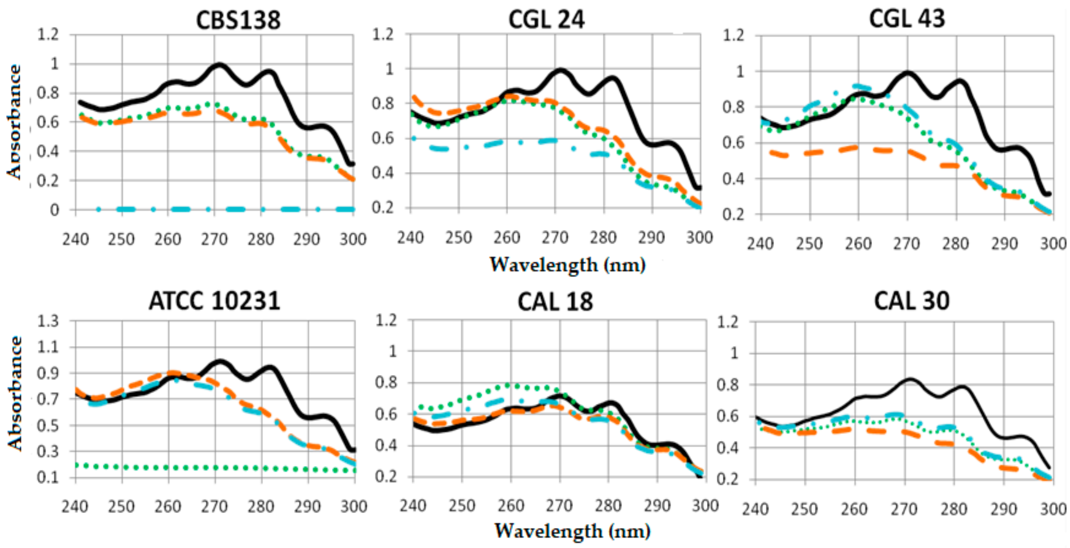

Figure S15. Reduction in ergosterol levels with simvastatin. The strains of *C. glabrata* (CBS, CGL24 and CGL43) and *C. albicans* (ATCC10231 CAL18 and CAL30) were grown in YNB without compound (black line) and with 100 µM of 2a (green line) , 2b (orange dashed line) and of 2c (blue dashed line). Followed by ethanolic extraction with KOH, the fraction corresponding to sterols was scanned from 240 to 300 nm. The quantities taken correspond to equal cell mass. The graph shows the absorbance values on the "x" axes and the wavelengths (nm) in the absorption spectrum on the "y" axes.

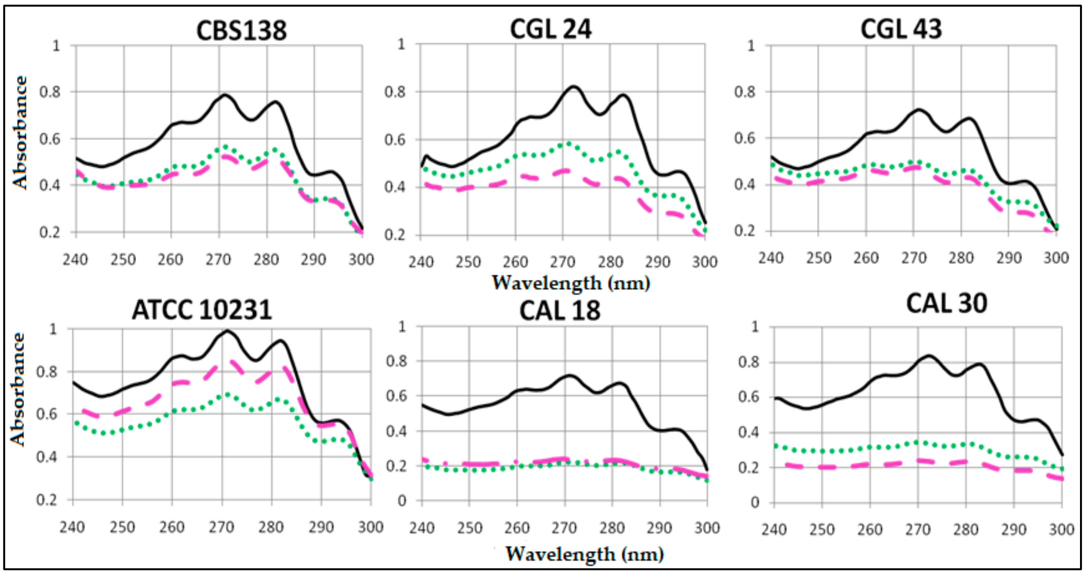

Figure S16. Reduction in ergosterol levels with simvastatin. The strains of *C. glabrata* (CBS, CGL24 and CGL43) and *C. albicans* (ATCC10231 CAL18 and CAL30) were grown in YNB without compound (black line) and with 100 µM of 3a (green line) and 3b (fushia dashed line). Followed by ethanolic extracción with KOH, the fraction corresponding to sterols was scanned from 240 to 300 nm. The quantities taken correspond to equal cell mass. The graph shows the absorbance values on the "x" axes and the wavelengths (nm) in the absorption spectrum on the "y" axes.

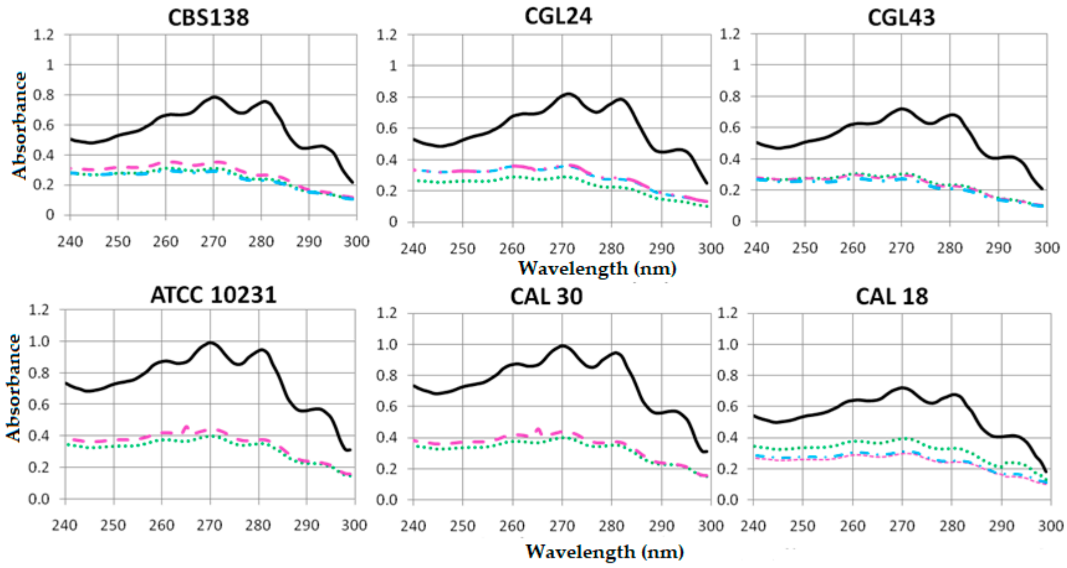

Figure S17. Reduction in ergosterol levels with simvastatin. The strains of *C. glabrata* (CBS, CGL24 and CGL43) and *C. albicans* (ATCC10231 CAL18 and CAL30) were grown in YNB without compound (black line) and with 100 μM of 4a (green dashed line), 4b (fushia dashed line) and 4c (blue dashed line). Followed by ethanolic extracción with KOH, the fraction corresponding to sterols was scanned from 240 to 300 nm. The quantities taken correspond to equal cell mass. The graph shows the absorbance values on the "x" axes and the wavelengths (nm) in the absorption spectrum on the "y" axes.

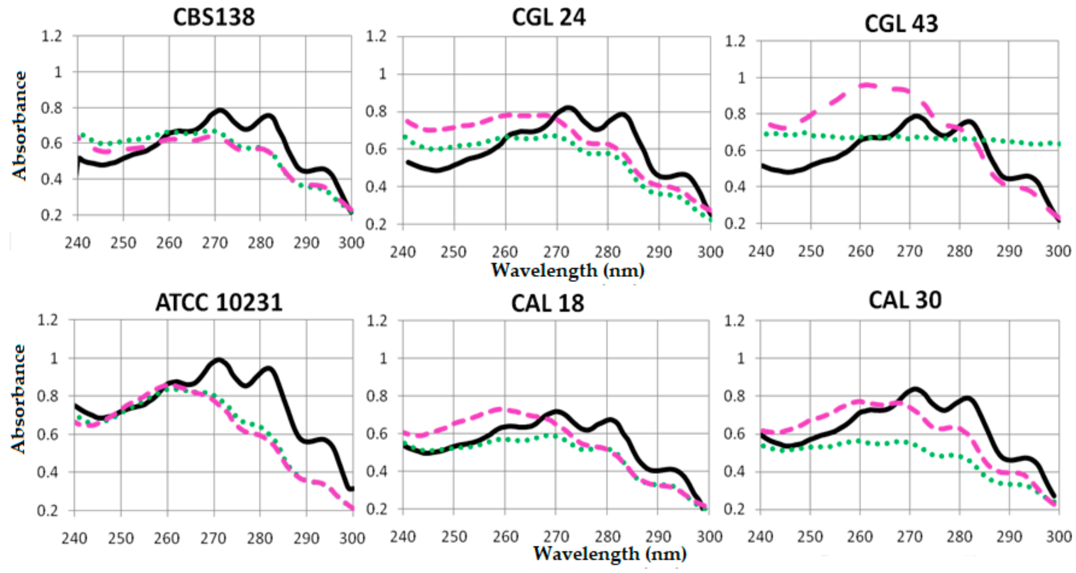

Figure S18. Reduction in ergosterol levels with simvastatin. The strains of *C. glabrata* (CBS, CGL24 and CGL43) and *C. albicans* (ATCC10231 CAL18 and CAL30) were grown in YNB without compound (black line) and with 100 μM of 5a (green dashed line) and 5b (fushia dashed line). Followed by ethanolic extracción with KOH, the fraction corresponding to sterols was scanned from 240 to 300 nm. The quantities taken correspond to equal cell mass. The graph shows the absorbance values on the "x" axes and the wavelengths (nm) in the absorption spectrum on the "y" axes.

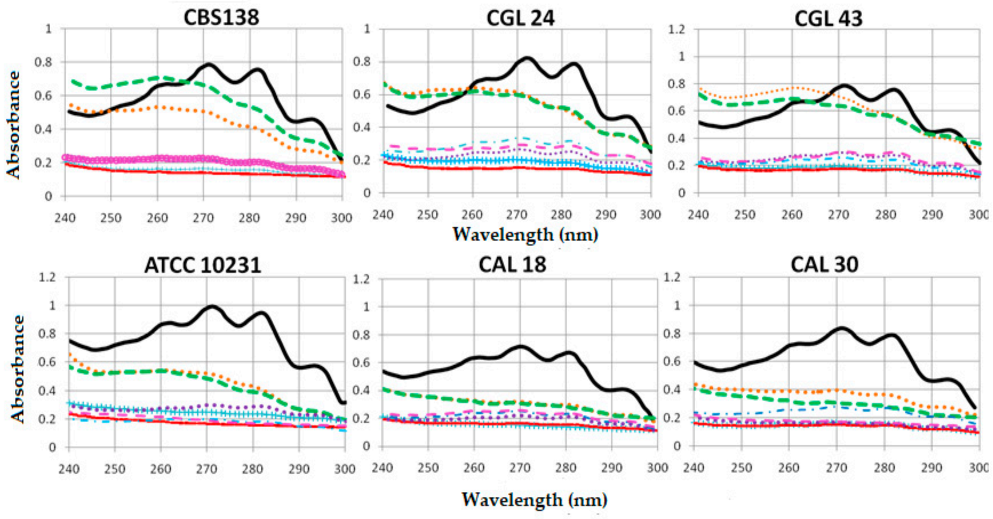

Figure S19. Reduction in ergosterol levels with simvastatin. The strains of *C. glabrata* (CBS, CGL24 and CGL43) and *C. albicans* (ATCC10231 CAL18 and CAL30) were grown in YNB without compound (black line) and with 100 µM of 6a (orange dashed line), 6b (green dashed line), 6c (light blue dashed line), 6d (red dashed line), 6e (purple dashed line), 6f (dark blue dashed line) and 6g (fushia dashed line). Followed by ethanolic extracción with KOH, the fraction corresponding to sterols was scanned from 240 to 300 nm. The quantities taken correspond to equal cell mass. The graph shows the absorbance values on the "x" axes and the wavelengths (nm) in the absorption spectrum on the "y" axes.

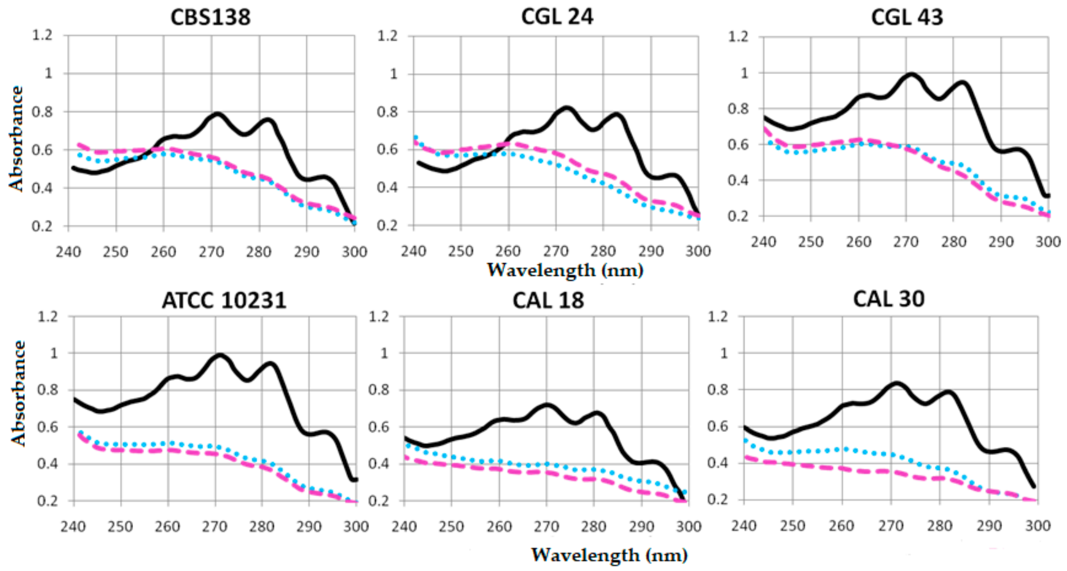

Figure S20. Reduction in ergosterol levels with simvastatin. The strains of *C. glabrata* (CBS, CGL24 and CGL43) and *C. albicans* (ATCC10231 CAL18 and CAL30) were grown in YNB without compound (black line) and with 100 µM of 7a (blue dashed line) and 7b (fushia dashed line). Followed by ethanolic extracción with KOH, the fraction corresponding to sterols was scanned from 240 to 300 nm. The quantities taken correspond to equal cell mass. The graph shows the absorbance values on the "x" axes and the wavelengths (nm) in the absorption spectrum on the "y" axes.

Table S1. Calculated physicochemical properties of simvastatin, fluconazole, a-asarone, and the series of test compounds 1a–b, 2a–c, 3a–b, 4a–c, 5a–b, 6a–g, and 7a–b.

Compound	MW(g/mol)	Log P	Log S	HA	HD
**Simvastatin**	418.572	4.4608	-4.75	5	1
**Fluconazole**	306.275	-0.1089	-2.175	7	1
**a-asarone**	212.288	1.6008	-1.545	3	0
**1a**	283.279	1.3698	-2.788	7	0
**1b**	254.281	1.9457	-2.032	5	1
**2a**	193.245	1.9329	-2.636	3	1
**2b**	194.229	2.2645	-2.264	3	1
**2c**	212.675	3.2162	-3.296	2	0
**3a**	238.326	3.5723	-2.983	3	0
**3b**	224.299	3.1179	-2.713	2	0
**4a**	236.266	2.1059	-2.267	4	0
**4b**	267.236	0.7564	-2.599	7	1
**4c**	250.293	2.5122	-2.567	4	0
**5a**	345.825	4.0878	-4.337	4	1
**5b**	345.825	4.15	-4.533	4	1
**6a**	194.229	1.8977	-2.181	3	0
**6b**	180.202	1.4914	-1.881	3	0
**6c**	194.229	1.907	-2.04	3	0
**6d**	208.256	2.3133	-2.34	3	0
**6e**	194.229	1.907	-2.04	3	0
**6f**	222.239	1.425	-2.521	4	0
**6g**	253.253	1.3917	-2.8	6	0
**7a**	196.245	2.3878	-2.129	3	1
**7b**	194.220	2.2023	-2.068	3	1

MW, molecular weight; Log P, partition coefficient; Log S, water solubility; H-A, hydrogen-bond acceptors and H-D, hydrogen-bond donors.

Table S2. Determination of the respiratory capacity of *petite* mutants of *C. glabrata* y *C. albicans* in YPD, YPG and YNB + glycerol. Presence or absence of mitochondrial genes.

Lane	Origin	Growth			Fragment (pb)	Loss DNAmt
		YPD	YPG	YNB + Glycerol		
1	Ca**6f**	+	-	-	-	Total
2	Ca**6f**	+	-	-	-	Total
3	Ca**4c**	+	+	+	1250	Partial
4	Ca**6g**	+	-	-	-	Total
5	Ca**6b**	+	+	+	-	Partial
6	Ca**6b**	+	-	-	-	Total
7	Ca**6b**	+	+	+	-	Partial
8	Ca**3a**	+	+	+	-	Partial
9	Ca**5a**	+	+	+	1250, 1000	Partial
10	Ca**5a**	+	+	+	1250, 1000	Partial
11	Cg**2b**	+	+	+	-	Total
12	Cg**6d**	ND	ND	ND	-	ND
13	Cg**7a**	+	+	-	-	Total
14	Cg**2b**	+	+	+	+	No loss
16	Cg**4b**	ND	ND	ND	-	ND
17	Cg**Sim**	+	-	-	1500, 1250, 1000	Partial
18	Cg**7a**	+	-	-	-	Total
19	Cg**3b**	+	-	-	-	Total
20	Cg**7a**	+	-	-	1500, 1250, 1000	Parcial
21	Cg**2b**	+	+	+	+	No loss
22	Cg**6f**	+	+	+	+	No loss
23	Cg**Sim**	+	-	-	-	Total
24	Cg**Sim**	ND	ND	ND	-	ND
25	Cg**Sim**	+	-	-	1500	Partial
26	Cg**Sim**	+	-	-	1500, 1250, 1000	Partial
27	Cg**Sim**	+	-	-	1500, 1250	Partial
15	CBS138	+	+	+	1500, 1250, 1000	No loss
28	ATCC10231	+	+	+	1500, 1250, 1000	No loss

+; growth, -; no groth, ND; undetermined
